# *Ginkgo biloba* Extract in Alzheimer’s Disease: From Action Mechanisms to Medical Practice

**DOI:** 10.3390/ijms11010107

**Published:** 2010-01-08

**Authors:** Chun Shi, Jun Liu, Fengming Wu, David T. Yew

**Affiliations:** 1 Department of Anatomy, Zhongshan School of Medicine, Sun Yat-Sen University, Guangzhou, Guangdong 510080, China; E-Mail: niaoeryaofei@163.com (F.W.); 2 Department of Anatomy, the Chinese University of Hong Kong, Shatin, N.T., Hong Kong, China; E-Mail: david-yew@cuhk.edu.hk (D.T.Y.); 3 Department of Neurology, the Second Affiliated Hospital, Sun Yat-sen University, Guangzhou, Guangdong 510120, China; E-Mail: docliujun@hotmail.com (J.L.)

**Keywords:** *Ginkgo biloba* extract, AD, neuroprotection

## Abstract

Standardized extract from the leaves of the *Ginkgo biloba* tree, labeled EGb761, is one of the most popular herbal supplements. Numerous preclinical studies have shown the neuroprotective effects of EGb761 and support the notion that it may be effective in the treatment and prevention of neurodegenerative disorders such as Alzheimer’s disease (AD). Despite the preclinical promise, the clinical efficacy of this drug remains elusive. In this review, possible mechanisms underlying neuroprotective actions of EGb761 are described in detail, together with a brief discussion of the problem of studying this herb clinically to verify its efficacy in the treatment and prevention of AD. Moreover, various parameters e.g., the dosage and the permeability of the blood brain barrier (BBB), impacting the outcome of the clinical effectiveness of the extract are also discussed. Overall, the findings summarized in this review suggest that, a better understanding of the neuroprotective mechanisms of EGb761 may contribute to better understanding of the effectiveness and complexity of this herb and may also be helpful for design of therapeutic strategies in future clinical practice. Therefore, in future clinical studies, different factors that could interfere with the effect of EGb761 should be considered.

## 1. Introduction

**Figure 1.**
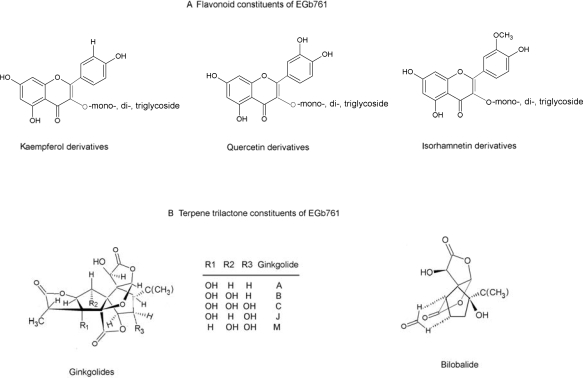
(A) Chemical structure of some representative flavonoid-*O*-glycosides and (B) terpene trilactone constituents of EGb761.

The leaves of the *Ginkgo* tree have a long history of being used for medicinal purposes. In the early 1970s, Dr. Willmar Schwabe Pharmaceuticals (Karlsruhe, Germany) successfully improved methodical procedures for the extraction and standardization of *Ginkgo biloba* preparation and produced highly concentrated and stable extracts from *Ginkgo biloba* leaves [1]. The extract of *Ginkgo biloba* leaves (EGb761) since then has been standardized to contain 24% flavonoid glycosides (containing quercetin, kaempferol, isorhamnetin *ect.*), 6% terpenoids (in which 3.1% are ginkgolides A, B, C, and J and 2.9% is bilobalide), and 5–10% organic acids (Figure 1). The flavonoids and terenoids are suggested to be the pharmacologically active constituents of EGb761 [2,3]. The organic acids in the extract contribute to its water solubility [4]. So far, numerous preclinical studies evaluating EGb761 effects have been undertaken and suggest the neuroprotective effects of this herb [5–11]. Nowadays, EGb761 has been widely used in the treatment and prevention of neurodegenerative dementias associated with ageing, Alzheimer’s Disease (AD), peripheral vascular diseases, and neurosensory problems (e.g., tinnitus) [8,12,13].

AD is one of the most common neurodegenerative disease associated with progressive cognitive and memory loss. The major molecular hallmarks of this disorder include extracellular deposition of the amyloid β peptide (Aβ) in senile plaques, intracellular neurofibrillary tangles, cholinergic deficit and neuronal loss in brain regions critical for memory and cognition [14–17]. A number of possible mechanisms for the pathological changes observed in AD brains include oxidative stress, inflammation, excitotoxicity and neuronal apoptosis [14,18–23]. Several protective agents such as antioxidants, anti-inflammatory drugs, cholinergic agents, estrogens, neurotrophic factors and calcium ion channel antagonists have been proposed for the prevention and treatment of AD, but none of them is proved to have a definite therapeutic effect [14,15,24–30]. In contrast, EGb761 has long been thought to be “multivalent” [7] and demonstrated to possess different profiles of action of various commonly-used drugs proposed for AD [30]. EGb761 thus has the potential to treat or prevent AD.

## 2. Mechanism of Neuroprotection by EGb761 and Its Constituents

### 2.1. Antioxidant Activity

Oxidative stress has long been thought to play a major role in the pathogenesis of AD [18]. The proposal that the beneficial action of EGb761 is mainly due to its free-radical scavenging action is supported by numerous *in vitro* and *in vivo* studies [7]. For example, pretreating cerebellar granule cells with EGb761 effectively attenuated oxidative damage triggered by H_2_O_2_/FeSO_4_ [31]. In another study using two AD models, Aβ-expressing neuroblastoma cell lineN2a and Aβ-expressing transgenic Caenorhabditis elegans, EGb761 was found to be able to attenuate the basal as well as the induced levels of H_2_O_2_-related reactive oxygen species (ROS) [7,32].

In addition to direct attenuation of ROS, EGb761 may also stabilize the cellular redox state by up-regulation of the protein level and activity of antioxidant enzymes [5]. For example, EGb761 was found to be able to increase the protein level and activity of superoxide dismutase (SOD) and catalase in rat hippocampus and rat ileum [5,33,34]. Moreover, activity of glutathione (GSH) reductase and gamma-glutamylcysteinyl synthetase, two enzymes critical for reduction and synthesis of GSH, were also enhanced by EGb761 [5,35,36].

The flavonoid fraction is suggested to be mainly responsible for the antioxidant properties of EGb761. It is proposed that, the flavonoid fraction evokes antioxidant effects *via* direct scavenging ROS, chelating prooxidant transitional metal ions and increase in antioxidant proteins such as SOD and GSH [7,37–40]. The polyphenol structure of flavonoids (Figure 1A) is thought to be responsible for their antioxidant actions [7,41]. Quercetin [42] and myricetin, two flavonoid constituents with such structure, especially effectively inhibit oxidation of *tert*-butylhydroperoxide [5]. In contrast, the antioxidant activity of terpene lactones is still in dispute [5]. There are conflicting data regarding the superoxide scavenging activity of bilobalide and the ginkgolides B, C and J [5,43,44]. Ginkgolide A has been demonstrated to lack the ability to scavenge the superoxide [5]. The discrepancy on the antioxidant activity of the terpene lactones may be explained as being due to differences in the type of oxidative stress used as well as the experimental models [5].

### 2.2. Protective Effects on Mitochondrial Function

Abnormalities in mitochondrial function are suggested to be associated with the pathological changes seen in AD [45]. Recently, EGb761 has been proposed to have direct protective effects on mitochondria. This may also contribute to its antioxidant effects, as mitochondrial respiratory chain is both the major target and the major source of ROS. Using SH-SY5Y cells, we reported that, EGb761 prevented amyloid β peptide (Aβ)-induced mitochondrial dysfunction, and thus reduced intracellular ROS generation [3]. This protective effect was observed with ginkgolide B but not with quercetin [3]. In another study using mitochondria isolated from PC12 cells, EGb761 and bilobalide up-regulated the gene expression of mitochondrial NADH dehydrogenase and decreased stage 4 respiration, whose increase is indicative of oxidative damage in mitochondria [46]. Protective effects of EGb761 on mitochondrial functions have also been demonstrated in *in vivo* studies. Using two animal models of aging, the senescence accelerated prone 8 mouse strain (SAMP8) and ovariectomized rats, we recently reported that EGb761 treatment effectively prevented the decrease of cytochrome c oxidase (COX) activity, mitochondrial ATP content and mitochondrial GSH content in hippocampi of aged SAMP8 mice and ovariectomized rats [47,48]. Despite these evidences, mechanisms underlying the protective effects of EGb761 and its constituents on mitochondrial function are still unclear and necessitate further studies.

### 2.3. Anti-Apoptotic Effect

Apoptosis has been implicated in the pathogenesis of various neurodegenerative diseases like AD [7,23,49]. As summarized by Smith and Luo in a review, the anti-apoptotic actions of EGb761 are multifactorial and may act synergistically upon multiple intracellular signaling pathways involved in apoptosis [4,7]. As for possible mechanisms underlying its anti-apoptotic action, EGb761 may maintain the integrity of the mitochondrial membrane; prevent cytochrome c release from the mitochondria, thereby blocking the formation of the apoptosome and the apoptotic caspase cascade; enhance the transcription of antiapoptotic Bcl-2-like protein; attenuate the transcription of pro-apoptotic caspase-12; inactivate pro-apoptotic *c-Jun* N-terminal kinase (JNK), thereby “turning off” downstream target *c-Jun*; inhibit the cleavage of the key effector protease caspase-3, thereby blocking the execution of apoptosis and prevent nuclear DNA fragmentation, the molecular hallmark of apoptosis [3,7,50].

The flavonoid fraction of EGb761 may be partly responsible for its anti-apoptotic properties. Despite the evidence that flavonoids prevent cell apoptosis induced by various oxidants [37–41], beneficial effects of EGb761 may go beyond its free-radical scavenging properties. Recently, evidences have accumulated to show that anti-apoptotic effects of flavonoids may be associated with modulation of specific proteins central to intracellular apoptotic signaling cascades such as the mitogen-activated protein kinase (MAPK) cascade. Quercetin, one of the major flavonoid constituents in EGb761 for example, did not inhibit JNK activity and apoptosis induced by hydrogen peroxide and 4-hydroxy-2-nonenal [51–53]. It is proposed that, quercetin exerts its anti-apoptotic effects by inactivation of the peroxide-induced JNK-*c-Jun*/AP-1 pathway and extracellular signal-regulated kinase (ERK)-*c-Fos*/AP-1 pathway [53,54]. But low concentrations of quercetin could also promote cellular survival by activation of the MAPK pathway (ERK2, JNK1, and p38), leading to expression of downstream survival genes (*c-Fos*, *c-Jun*) and defensive genes (phase II detoxifying enzymes; GSH *S*-transferase, quinone reductase) [55].

The terpene fraction of EGb761 may also contribute to its anti-apoptotic properties. Bilobalide, ginkgolide B, and ginkgolide J were demonstrated to be able to attenuate apoptosis in chick embryonic neurons caused by 24-h exposure to serum deprivation [56,57]. Bilobalide could also reverse apoptotic damage induced by 12-h staurosporine treatment of chick neurons [57]. In mixed cultures of neurons and astrocytes from neonatal rat hippocampus, bilobalide rescued the neurons from serum deprivation-induced apoptosis, and both bilobalide and ginkgolide B attenuated staurosporine-triggered apoptotic damage [57]. However, ginkgolide A failed to block apoptotic damage either in serum-deprived or in staurosporine-treated neurons [58]. Analysis of DNA fragmentation and the activities of caspase-1- and caspase-3- like protease suggest that bilobalide can block neuronal apoptosis in the early stage by attenuating the elevations of c-myc, p53, and Bax and the activation of caspase-3 [57]. Similarly, the anti-apoptotic effects of ginkgolides are also suggested to be associated with blockage of early signaling events in apoptosis. A recent study demonstrated that ginkgolide B inhibited ethanol-induced apoptotic cell death *via* suppression of activation of JNK and caspase 3 [59]. Our recent study supported this finding by showing that ginkgolide B protected against Aβ-induced activation of JNK, ERK1/2 and Akt signaling pathways and DNA fragmentation in SH-SY5Y cells [3]. However, there are also contrasting data showing that the terpene constituents of EGb761 have no protective effect on cell apoptosis. For example, bilobalide failed to protect primary adult rat hippocampal neurons against apoptosis caused by a peroxyl radical-generator, 2,2′-azobis-2-amidinopropane [57,60]. In another study, the terpenes of EGb761 could not block hydroxyl radical-induced apoptosis in rat cerebellar granule cells [61]. This discrepancy may be due to the different types of cells and different methods for inducing apoptosis [57].

Despite the anti-apoptotic effects of EGb761 and its pharmacologically active components, the pro-apoptotic action of EGb761 and its constituents (e.g., quercetin and ginkgolide B) has also been demonstrated [50]. Treatment dosage may be one of the vital factors that determine the specific action of EGb761 and its constituents on apoptosis [50] (discussed below).

### 2.4. Anti-Inflammatory Effect

Inflammation has been implicated in the pathology of AD [62]. Cytokines, acute phase reactants, and other inflammatory mediators have been found to be up-regulated in pathologically vulnerable regions of AD brains [62]. EGb761 has been demonstrated to have anti-inflammatory effects [63–65]. These effects may be attributed to the combined actions of its ginkgolide and flavonoid constituents [65].

The anti-inflammatory action of ginkgolides may be associated with their platelet-activating factor (PAF) -antagonist activity. Substantial evidence suggests the role of PAF as a regulator of cytokines in inflammatory responses [4,66]. Intracerebroventrical administration of PAF in rats could stimulate the synthesis of pro-inflammatory mediator leukotriene, particularly leukotriene C4 [4,67]. PAF can be synthesized in neurons following stimulation with neurotransmitters such as *N*-methyl-d-aspartic acid (NMDA) and glutamic acid and plays various roles in neuronal functions and brain development [68]. However, increased concentrations of PAF in the brain are also implicated in neurodegenerative diseases such as AD [69,70]. Ginkgolides display very specific and potent antagonist effects against PAF [65]. Intracerebroventrical administration BN-52021 (ginkgolide B) in rats significantly attenuated PAF-induced rise in cerebrospinal fluid peptidoleukotriene levels [4,67]. BN-52021 could also reduce PAF-induced production of the eicosanoid and thromboxane B in a fetal rat brain [4,71]. In support of these findings, our recent study has shown that ginkgolide B could completely block PAF-induced decrease of cell *via*bility in SH-SY5Y cells [3]. In addition to PAF-antagonizing activity, the inhibitory effects of ginkgolides A and B on pro-inflammatory cytokines tumour necrosis factor-alpha and interleukin-1 production in lipopolysaccharide-stimulated rat microglial cultures were also observed [4,72].

On the other hand, the flavonoid fraction of EGb761 reportedly inhibits lipooxygenase that is concerned with the formation of leukotrienes [65]. In addition, in one of our recent studies, we have demonstrated that, a flavonoid constituent of EGb761, quercetin may also be involved in EGb761’s PAF-antagonist activity [3].

### 2.5. Protective Effects against Amyloidogenesis and Aβ Aggregation

The accumulation of Aβ plaques has been proposed to be one of the most prominent mechanisms underlying the pathology of AD [73]. Recently, the role of EGb761 in the protection against the Aβ-induced toxicity has received much attention. A number of recent reports indicate that EGb761 protects against Aβ-induced neurotoxicity by blockage of Aβ-induced events, such as ROS accumulation, glucose uptake, mitochondrial dysfunction, activation of AKT, JNK and ERK 1/2 pathways and apoptosis [3,74,75]. In addition to the protective effects against Aβ, EGb761 has also been shown to prevent amyloidogenesis [76–79]. On hippocampal slices, Colciaghi *et al*. demonstrated that EGb761 could push amyloid precursor protein (APP) metabolism towards the α-secretase pathway, thereby increasing the release of the soluble form of APP (sAPPα) [76,78]. Using the transgenic AD model Tg2576 mice, the consequence of the capability of EGb761 to influence positively the α-secretase pathway was also assessed *in vivo*. [78,79]. It was found that, after EGb761 treatment, Tg-2576 mice exhibited an enhancement of spatial learning and memory comparable to wild type mice [78,79]. It has been proposed that, EGb761 inhibits the production of brain Aβ by lowering the levels of circulating free cholesterol, as free circulating and intracellular cholesterol levels could affect APP processing and amyloidogenesis [77,78,80–82]. Despite these evidences, further investigations are needed to identify the major constituents responsible for this antiamyloidogenic effect.

EGb761 could also inhibit the formation of Aβ fibrils [78,86]. It is known that, the β-sheet structure of Aβ fibrils is mainly responsible for the neurotoxicity of Aβ and may also help Aβ escape from clearance *via* proteolytic degradation [78,83–85]. Thus, inhibiting of formation of β-sheet structure of Aβ fibrils may also help to prevent Aβ toxicity. The interaction of Aβ with transition metal ions, notably iron, zinc and copper, could influence the aggregation state of Aβ. EGb761, through its iron chelating property, may inhibit Aβ fibrils formation [78,86]. EGb761 may also influence the formation of Aβ fibrils by increasing gene expression of transthyretin [87], as transthyretin has been shown to prevent Aβ aggregation *in vitro* by sequestering Aβ monomers [88]. The inhibitory effect of EGb761 on Aβ aggregation was also observed with bilobalide, ginkgolide J and flavonoid compounds [78,86].

### 2.6. Other Mechanisms

Other mechanisms which may be involved in neuroprotective effects of EGb761 on AD are ion homeostasis, modulation of phosphorylation of tau protein, and induction of growth factor synthesis.

Ca^2+^ dyshomeostasis may be of pivotal importance in mediating neutotoxicity in AD [89,90]. Our unpublished data suggested that, EGb761 could protect against Aβ (1–42)-triggered Ca^2+^ influx *via N*-methyl-d-aspartic acid receptors. This effect was also observed with its constituents quercetin and ginkgolide B.

In AD brain, hyperphosphorylated microtubule-associated protein tau is aggregated as neuro-fibrillary tangles of paired helical filaments, which is a key event in the pathogenesis of AD [5,91]. Using mRNA microarrays, Watanabe *et al*. found that EGb761 was able to up-regulate gene expression of microtubuli-associated tau protein as well as of neural protein phosphatase type 1, a serine/threonine protein phosphatase known to dephosphorylate hyperphosphorylated tau protein, in the hippocampus and cortex of normal mice [5,87].

Altered levels of nerve growth factor (NGF) have been detected in AD brains [92–94]. EGb761 was reported to be able to up-regulated mRNA expression of NGF such as growth hormone and prolactin in mouse cortex [5,94]. Furthermore, the mRNA and protein expression of glial-derived neurotrophic factor and vascular endothelial growth factor in cultured rat cortical astrocytes could also be up-regulated by a terpenoid constituent of EGb761, bilobalide [5,95].

## 3. Current Status of Clinical Use of EGb761: Still a Long Way from Preclinical Promise

Despite the substantial body of preclinical evidence suggesting that EGb761 may be effective in treatment and prevention of AD, its clinical effect remains elusive. A recent meta-analysis of clinical studies (925 AD patients in 9 trials) on the use of EGb761 suggested that beneficial effects of EGb761 in AD are inconsistent [96]. In addition, the *Ginkgo* Evaluation of Memory (GEM) study, the largest and longest randomized controlled trial of *Ginkgo biloba* extract concluded that EGb761 supplement was not effective in reducing either the overall incidence rate of dementia or AD incidence in elderly individuals with normal cognition or those with mild cognitive impairment [97]. These reports suggest to us that, despite the preclinical promise that EGb761 may be effective in treatment and prevention of AD, this promise has not completely translated to clinical research benefits.

Certain confounding factors, as previously suggested [1], may be interfere with EGb761’s effect, and may be the source of the variations observed among EGb761 studies. First, the therapeutic effect of EGb761 may depend on the sensitivity of the study population to this drug. Thus the homogeneity of the study population may affect EGb761’s efficacy [1]. In two multicenter trials, EGb761 treatment showed a greater effect on the AD subgroup than the total mixed dementia population (AD and Multi-Infarct Dementia) [1]. Moreover, it is still unclear whether age and sex influence population sensitivity to EGb761 treatment. Second, the therapeutic effect of EGb761 in AD may be associated with the severity of the impairment [1]. In the group of patients with very mild to mild cognitive impairment, EGb761 effect could be considered in the term of improvement while in more severe dementia, EGb761 effect should be considered more in terms of stabilization or slowing down of worsening [1,80]. Third, the sensitivity of different cognitive outcome measurements such as the Syndrom-Kurztest and the Alzheimer’s Disease Assessment Scale-Cognitive Subscale may affect the assessment of EGb761’s effect, but this factor may be directly dependent on the severity of the cognitive impairment of the tested patients [1]. Last but not least, there may be a dose-dependent effect of EGb761. Nowadays, a daily dose of 240 mg has been extensively used to stabilize the disease progression in patients with AD [40,77,98,99]. But whether doses higher than 240 mg/day further enhances the effect of EGb761 remains unclear [1].

Regarding these factors, basic researches gives useful information that may be helpful for modifying the clinical efficacy of EGb761. In one of our recent studies, we investigated the dosage effects of EGb761 on H_2_O_2_-induced apoptosis in human neuroblastoma SH-SY5Y cells [50]. We found that, low doses of EGb761 (50–100 μg/mL) inhibited H_2_O_2_-induced cell apoptosis *via* inactivation of Akt, JNK and caspase 3 while high doses of EGb761 (250–500 μg/mL) enhanced H_2_O_2_ toxicities *via* inactivation of Akt and enhancement of activation of JNK and caspase 3 [50]. We also found that, H_2_O_2_ decreased intracellular GSH content, which was also inhibited by low concentrations of EGb761 but enhanced after high concentrations of EGb761 treatment [50]. These results suggest that, EGb761 has dosage-dependent effects on H_2_O_2_-induced cell apoptosis, which may be correlated with regulation of cell redox state. In support of these findings, previous studies have demonstrated similar dosage effects of quercetin and ginkgolide B, two major components of EGb761 [53,59]. In addition, most of protective effects of EGb761 observed *in vitro* were reported at lower concentrations (≤200 μg/mL) [31,36,46,56,61,74,77,86,100]. Therefore, only within a certain range of dosage, EGb761 shows protective effects.

EGb761 consists of hundreds of chemical constituents [77]. As ginkgolide constituents of EGb761 represent approximately 2–3%, these compounds were recently used to monitor its bioavailability *in vivo* [77,101]. In one of our recent studies, we have demonstrated that, EGb761 at 100 μg/mL could protect against cell death induced by a wide range of H_2_O_2_ doses (250–1,000 μM) [50]. Similar doses have been reported in *in vivo* studies. It has been demonstrated that, after oral administration of 240 mg/day of EGb761 to human subjects or 100 mg/kg EGb761 in rats, the concentration of ginkgolides in the blood reached 2–3 μg/mL, a concentration suggesting the presence of 100 μg/mL EGb761 in the blood [77,102]. However, even if protective concentrations of EGb761 are achieved in the blood, for the pharmacologically active components of EGb761 to modulate central mechanisms, they have to cross the BBB and accumulate in the brain. It is known that, under normal physiological conditions, the BBB controls tightly the entry of drugs into the central nervous system. But disruption of BBB is now increasingly documented in both normal ageing and neurodegenerative disorders such as AD [103,104]. It has been purported that, under pathological conditions such as AD, EGb761 was able to cross the BBB effectively and retain its neuroprotective properties [105,106], but there is a lack of evidence for the ability of EGb761 to cross the BBB under normal physiological conditions. Thus BBB permeability may be an important factor that interferes with the *in vivo* effects of EGb761 and even determines population differences in sensitivity to EGb761 treatment. In order to better understand the *in vivo* pharmacological actions of EGb761, we have used some animal models of aging (e.g., SAMP8 mice and ovariectomized rats) to test and compare effects of EGb761 on mitochondrial function in platelets and central nervous system. The rationale of employing platelet as a peripheral biomarker for mitochondrial damages rests on the assumption that alterations occurring in ageing and age-associated disorders may be present in all cells, and that changes occurring in platelet mitochondria may represent generalized bioenergetic deficiencies [107].

Epidemiological studies have shown that the incidence of female AD increases significantly after menopause by two to three times that of males, suggesting that estrogen withdrawal may play a primary role in the pathogenesis of AD in post-menopausal women [14,108]. Using ovariectomized middle-aged rats to mimic the post-menopausal pathophysiological changes in women, we demonstrated that, oral administration of 100 mg/kg EGb761 protected against the decrease of COX activity, mitochondrial ATP content and mitochondrial GSH content in both platelets and hippocampi, suggesting its peripheral and central effects against estrogen withdrawal-induced degeneration [47]. In contrast, in sham-operated rats, EGb761 increased mitochondrial GSH content in platelets but failed to show similar effect on hippocampi, indicating that EGb761 may help to enhance the functional reserve of mitochondria, but this effect was limited to the outside of the central nervous system [47]. Previous studies have demonstrated that estrogen withdrawal through ovariectomy increases the selective permeability of the BBB [109,110]. EGb761 displayed similar effects on platelets and hippocampi of ovariectomized rats but showed differential effects on platelets and hippocampi of sham-operated rats, possibly because estrogen withdrawal induced an increase of BBB permeability [47]. In another study using two age groups (3-week-old and 40-week-old) of SAMP8 mice, a model of age-related cognitive decline with relevance to genetic alterations and protein abnormalities in AD, we found that, oral administration of 100 mg/kg EGb761 protected against the decrease of COX activity, mitochondrial ATP content and mitochondrial GSH content in platelets of young and old mice, suggesting the peripheral effect of this herb in the prevention and treatment of age-associated degeneration [48]. In contrast, in hippocampi, protective effects of EGb761 were observed only in the old mice, probably due to an age-associated increase in the permeability of the BBB [48]. From these data, we speculate that, EGb761’s effect may be limited to the outside of the central nervous system under normal physiological conditions, but increased BBB permeability may enhance the central effect of EGb761. If so, future studies are needed to determine the ability of pharmacologically active components of EGb761 to cross the BBB and various methods that help to deliver them across the intact BBB may contribute to enhancement of EGb761’s efficacy in the prevention of AD.

In addition, improvement of neuronal survival environment may also help to enhance neuronal sensitivity to EGb761. In fact, a number of aging-related neurotoxic events, such as oxidative stress and decreased neuronal trophic support by neurotrophic factors may co-exist to perturb neuronal physiological homeostasis *in vivo* during ageing of the central nervous system or the development of age-related neurodegenerative diseases such as AD [93,100]. Thus, interventions could be targeted accordingly or comprehensively [100]. In one of our recent studies, rat pheochromocytoma (PC12) cells were treated with H_2_O_2_ for 24 h to reduce intracellular GSH content to about 50% [100]. After withdrawal of H_2_O_2_, we determined effects of EGb761 on cell death under three different conditions of serum supply: normal growth medium, serum deprivation and serum deprivation followed by re-supply [100]. We found that, under the condition of serum deprivation, the percentage of dead cells is lower than under the condition of serum supply, partially *via* inhibition of mitochondrial metabolism [100]. Moreover, after serum deprivation, serums re-supply exacerbated cell necrosis, possibly through enhancement of oxidative damage [100]. EGb761 protected against cell death under the condition of serum supply whereas showed little protective effects on serum-depleted cells [100]. These results suggest that, there may be a synergistic effect between trophic factors and EGb761 [100]. EGb761 prevents cells from possible oxidative damage induced by the trophic factors [100]. On the other hand, trophic factors strengthen cellular responses to EGb761 treatment [100]. Therefore, a combined treatment of EGb761 and neurotrophic factors in future basic and clinical research is proposed.

Overall, these findings suggest that, to achieve a stable and satisfied clinical efficacy of EGb761, different factors that could interfere with the efficacy of this drug should be considered.

Despite our data suggesting that high doses of EGb761 may exacerbate cellular oxidative damage and apoptosis, no serious side effects of EGb761 have been noted in any trials so far [5]. Only mild gastrointestinal complaints, dizziness, headache, dry mouth, sleep disturbances, transient cyanosis of nails and lips, and allergic skin reactions have been reported in rare cases [5]. Moderate concentrations of EGb761 seem to be well tolerated [5].

## 4. Conclusions

Various *in vivo* and *in vitro* preclinical studies support the notion that standardized *Ginkgo biloba* extract EGb761 may be effective in the treatment and prevention of AD and other age-related, neurodegenerative disorders. Anti-oxidation, anti-apoptosis, anti-inflammation, protection against mitochondrial dysfunction, amyloidogenesis and Aβ aggregation, ion homeostasis, modulation of phosphorylation of tau protein and even induction of growth factors are possible mechanisms of action. However, the clinical efficacy of EGb761 still remains elusive. Multiple factors such as population sensitivity, severity of impairment, type of assessments used to measure efficacy and doses were suggested to be able to interfere with EGb761 efficacy in clinical practice. Regarding these factors, basic scientific reports give useful information that may help to modify the clinical efficacy of this drug. Overall, a better understanding of the mechanisms underlying neuroprotective effects of EGb761 may contribute to better understanding of the effectiveness and complexity of this drug, and may also be helpful for designing therapeutic strategies in future clinical practice.
